# Testicular dysgenesis/regression without campomelic dysplasia in patients carrying missense mutations and upstream deletion of *SOX9*

**DOI:** 10.1002/mgg3.165

**Published:** 2015-07-14

**Authors:** Yuko Katoh‐Fukui, Maki Igarashi, Keisuke Nagasaki, Reiko Horikawa, Toshiro Nagai, Takayoshi Tsuchiya, Erina Suzuki, Mami Miyado, Kenichiro Hata, Kazuhiko Nakabayashi, Keiko Hayashi, Yoichi Matsubara, Takashi Baba, Ken‐ichirou Morohashi, Arisa Igarashi, Tsutomu Ogata, Shuji Takada, Maki Fukami

**Affiliations:** ^1^ Department of Molecular EndocrinologyNational Research Institute for Child Health and DevelopmentTokyoJapan; ^2^ Division of PediatricsDepartment of Homeostatic Regulation and DevelopmentNiigata University Graduate School of Medical and Dental SciencesNiigataJapan; ^3^ Division of Endocrinology and MetabolismNational Center for Child Health and DevelopmentTokyoJapan; ^4^ Department of PediatricsDokkyo Medical University Koshigaya HospitalKoshigayaJapan; ^5^ Department of Maternal‐Fetal BiologyNational Research Institute for Child Health and DevelopmentTokyoJapan; ^6^ National Research Institute for Child Health and DevelopmentTokyoJapan; ^7^ Department of Molecular BiologyGraduate School of Medical SciencesKyushu UniversityFukuokaJapan; ^8^ Department of Systems BioMedicineNational Research Institute for Child Health and DevelopmentTokyoJapan; ^9^ Department of PediatricsHamamatsu University School of MedicineHamamatsuJapan

**Keywords:** Campomelic dysplasia, deletion, enhancer, mutation, testis

## Abstract

*SOX9* haploinsufficiency underlies campomelic dysplasia (CD) with or without testicular dysgenesis. Current understanding of the phenotypic variability and mutation spectrum of *SOX9* abnormalities remains fragmentary. Here, we report three patients with hitherto unreported *SOX9* abnormalities. These patients were identified through molecular analysis of 33 patients with 46,XY disorders of sex development (DSD). Patients 1–3 manifested testicular dysgenesis or regression without CD. Patients 1 and 2 carried probable damaging mutations p.Arg394Gly and p.Arg437Cys, respectively, in the *SOX9* C‐terminal domain but not in other known 46,XY DSD causative genes. These substitutions were absent from ~120,000 alleles in the exome database. These mutations retained normal transactivating activity for the *Col2a1* enhancer, but showed impaired activity for the *Amh* promoter. Patient 3 harbored a maternally inherited ~491 kb *SOX9* upstream deletion that encompassed the known 32.5 kb XY sex reversal region. Breakpoints of the deletion resided within nonrepeat sequences and were accompanied by a short‐nucleotide insertion. The results imply that testicular dysgenesis and regression without skeletal dysplasia may be rare manifestations of *SOX9* abnormalities. Furthermore, our data broaden pathogenic *SOX9* abnormalities to include C‐terminal missense substitutions which lead to target‐gene‐specific protein dysfunction, and enhancer‐containing upstream microdeletions mediated by nonhomologous end‐joining.

## Introduction

SOX9 (OMIM *608160) controls embryonic development by transactivating several genes such as *COL2A1* involved in skeletal formation and *AMH* involved in testicular development. Known *SOX9* mutations include various missense substitutions in the high‐mobility group or dimerization domains, as well as several nonsense, frameshift, and splice‐site mutations widely distributed in the coding region (Meyer et al. 1997; Bernard et al. 2003; Harley et al. 2003; Michel‐Calemard et al. 2004; Staffler et al. 2010). Patients with *SOX9* mutations manifest campomelia, hypoplastic scapulae, pelvic anomalies, micrognathia, and cleft palate, collectively referred to as campomelic dysplasia (CD), although a certain percentage of mutation‐positive patients show a mild variant of CD that lacks campomelia (acampomelic CD: ACD) (Bernard et al. 2003; Michel‐Calemard et al. 2004; Staffler et al. 2010). *SOX9* mutations also result in complete or partial gonadal dysgenesis in individuals with 46,XY karyotype (Meyer et al. 1997; Michel‐Calemard et al. 2004). As CD/ACD‐compatible skeletal abnormalities were described in all patients with *SOX9* mutations and disorders of sex development (DSD) were shared only by ~70% of 46,XY patients (Mansour et al. 1995), it seems that skeletal tissues are more vulnerable than testis to impaired SOX9 function. Kwok et al. (1996) suggested that *SOX9* mutations are unlikely to underlie 46,XY DSD in the absence of skeletal abnormalities.

Recent studies have identified submicroscopic deletions in the *SOX9* upstream region in six patients with isolated 46,XY DSD (Pop et al. 2004; Lecointre et al. 2009; Kim et al. 2015). These patients shared a 32.5 kb overlapping region of deletion at a position 607–640 kb upstream of the *SOX9* start codon, which was designated as the XY sex reversal region (XYSR). Since *SOX9* expression is regulated by multiple tissue‐specific enhancers (Bagheri‐Fam et al. 2006), XYSR likely contains a testis‐specific enhancer. Considering the limited number of reported patients, further studies are necessary to clarify the phenotypic variability and mutation spectrum of *SOX9* abnormalities. Furthermore, the genomic basis of *SOX9* upstream deletions remains to be investigated. Here, we report three unique cases with *SOX9* abnormalities.

## Materials and Methods

### Subjects

This study was approved by the Institutional Review Board Committee at the National Center for Child Health and Development. The study group consisted of 33 Japanese patients with 46,XY DSD. All patients showed genital abnormalities at birth; of these, 29 had isolated DSD, whereas the remaining patients manifested DSD with additional clinical features. Eleven and 22 patients were raised as a female and male, respectively. Patients with apparent chromosomal abnormalities were excluded from this study.

### Mutation analysis

After obtaining written informed consent from the patients or their parents, genomic DNA samples were collected from the patients. Mutation analysis was performed by next‐generation sequencing (NGS). Genomic DNA samples were isolated from peripheral leukocytes. Target regions in the human genome were amplified with the SureSelect Target Enrichment system (G7531C or all exome v5; Agilent Technologies, Palo Alto, CA) and sequenced on a HiSeq 2000 sequencer (Illumina, San Diego, CA). Nucleotide alterations were called by Avadis NGS 1.3.1 (DNA Chip Research, Yokohama, Japan) or SAMtools 0.1.17 software (http://samtools.soursefrge.net/). In this study, we focused on protein‐altering substitutions and splice‐site mutations of 27 known causative genes for 46,XY DSD, that is, *AKR1C2*,* AKR1C4*,* AMH*,* AMHR2*,* AR*,* ATF3*,* ATRX*,* BNC2*,* CYP11A1*,* DHH*,* DMRT1*,* GATA4*,* HSD3B2*,* HSD17B3*,* INSL3*,* INSR*,* LHCGR*,* MAP3K1*,* NR5A1*,* POR*,* RXFP2*,* SOX9*,* SRD5A2*,* SRY*,* STAR*,* TSPYL1*, and *WT1*. Nucleotide substitutions of allele frequency 1% or higher in the Japanese population (Human Genetic Variation Browser, http://www.genome.med.kyoto-u.ac.jp/SnpDB) were excluded as polymorphisms. *SOX9* (NM_000346.3) mutations indicated by NGS were confirmed by Sanger sequencing using a primer pair: SOX9‐exon3FW2 (5′‐CAGGCGCACACGCTGACCAC‐3′) and SOX9‐exon3RV (5′‐CCTCTCTTTCTTCGGTTAT‐3′). Furthermore, PCR products carrying the nucleotide alterations were subcloned into the TOPO TA cloning vector (Life Technologies, Carlsbad, CA) and the mutant and wild‐type alleles were sequenced separately. Whenever possible, parental samples of mutation‐positive patients were also subjected to molecular analysis.

### Functional analyses of *SOX9* substitutions

Conservation and functional consequences of *SOX9* substitutions were predicted using Polyphen‐2 (http://genetics.bwh.harvard.edu/pph2/) (Adzhubei et al. 2010). Population frequencies of the substitutions were analyzed using the Exome Aggregation Consortium Browser (http://exac.broadinstitute.org/).

The transactivating activity of the substitutions was assessed by a previously reported method with modifications (Kelberman et al. 2006). Briefly, an expression vector for wild‐type *SOX9* was purchased from Origene Technologies (RC208944, Rockville, MD), and each *SOX9* mutation was introduced into the expression vector by site‐directed mutagenesis (PrimeSTAR Mutagenesis Basal Kit; Takara Bio, Ohtsu, Japan). In this study, we compared the transactivating activity of newly identified mutants to that of the known ACD‐associated *SOX9* mutant c.527C>T (p.Pro176Leu) (Michel‐Calemard et al. 2004). We used a PGL3 reporter vector (Promega, Madison, WI) containing the murine *Amh* promoter sequence (from −231 to 0 to the transcription start site of *Amh*, NC_000076.6) and a PGL4 reporter vector (Promega) containing the murine *Col2a1* enhancer sequence (from +1958 to +2485 to the transcription start site of *Col2a1*, NC_000081.6). An expression vector for *NR5A1* was kindly provided by Professor Toshihiko Yanase (Fukuoka University, Fukuoka, Japan). Luciferase assays for the *Amh* promoter and for the *Col2a1* enhancer were carried out using COS‐1 (RIKEN, Ibaraki, Japan) and HEK293 cells (Health Science Research Resources Bank, Tokyo, Japan), respectively. The cells were seeded in 6‐well dishes and treated with Lipofectamine 2000 Reagent (Life Technologies). For *Amh* promoter assays, we transfected cells with 40 ng *SOX9* expression vector, 100 ng *NR5A1* expression vector, 1 *μ*g reporter vector, and 3 ng pCMV‐PRL control vector (Promega). For *Col2a1* enhancer assays, we used 200 ng *SOX9* expression vector, 500 ng reporter vector, and 3 ng pCMV‐PRL vector. At 48 h after transfection, luciferase activity was measured using the Dual‐Luciferase Reporter Assay System (Promega) with Lumat LB9507 (Berthold, Oak Ridge, TN). Every assay was performed in triplicate and all experiments were repeated at least three times. The results are expressed as the mean ± one standard deviation, and statistical significance was calculated by the Student *t* test. *P* values of <0.005 were considered significant.

### Copy‐number analysis

Copy‐number alterations were analyzed by comparative genomic hybridization using a catalog human array (4 × 180 k format) or a custom‐made array (design ID, 031687) (Agilent Technologies). The deletion breakpoints were determined by direct sequencing of PCR products harboring the fusion junction. The products were generated using a primer pair: 5′‐TTTTTTCCTTGAAGTTAATG‐3′ and 5′‐AATGTAGTGCTATATATTGC‐3′. Sizes and genomic positions of the deletions were analyzed using the UCSC genome browser (http://genome.ucsc.edu/; GRCh37/hg19) and the presence or absence of repeat sequences was examined with RepeatMasker (http://www.repeatmasker.org). We referred to the Database of Genomic Variants (http://projects.tcag.ca/variation/) to exclude known benign variants.

## Results

### Mutation analysis

**Figure 1:**
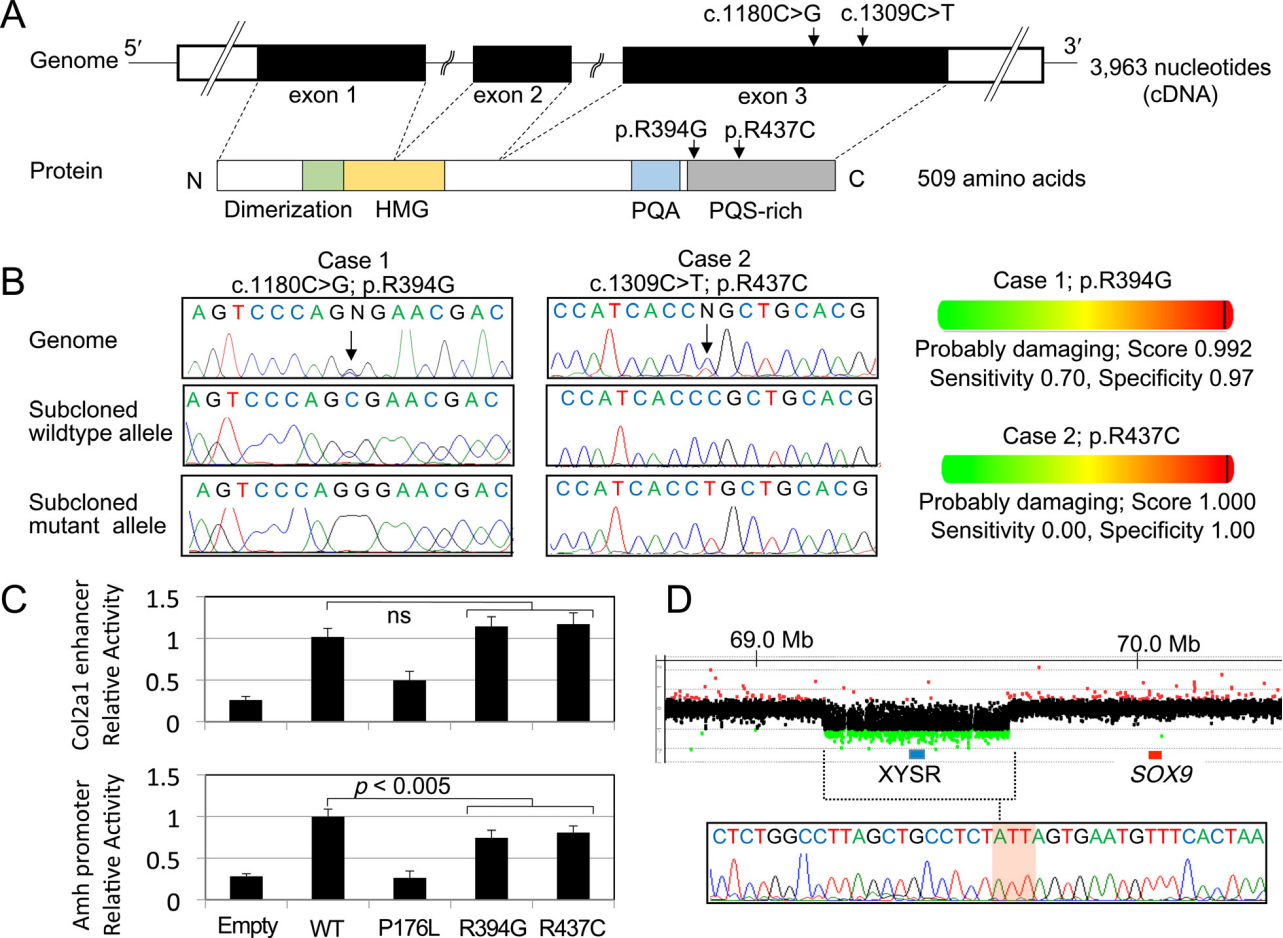
*SOX9* abnormalities in patients 1–3. (A) Genomic and protein structures of *SOX9*/SOX9. The positions of the c.1180C>G (p.Arg394Gly) and c.1309C>T (p.Arg437Cys) mutations are indicated by arrows. White and black boxes in the upper panel indicate the untranslated and coding regions, respectively. Colored boxes in the lower panel indicate dimerization (codon 60–101) (Bernard et al. 2003), high‐mobility group (HMG: codon 101–184), proline/glutamine/alanine (PQA: codon 339–379), and proline/glutamine/serine‐rich (PQS‐rich: codon 386–509) domains (McDowall et al. 1999). (B) Nucleotide substitutions detected in patients 1 and 2. Left panel: electro chromatograms of the mutations. The mutated nucleotides are indicated by arrows. Right panel: in silico functional prediction of mutant proteins. (C) In vitro assays using reporters containing the *Col2a1* enhancer or *Amh* promoter. The transactivating activity of p.Arg394Gly and p.Arg437Cys mutants was compared to that of the known ACD‐associated SOX9 mutant p.Pro176Leu (Michel‐Calemard et al. 2004). The results are expressed as the mean ± one standard deviation. Relative transactivating activities of the SOX9 mutants against the wild‐type are shown. Empty: empty expression vector; ns: not significant. (D) *SOX9* upstream deletion in patient 3. Upper panel: array‐based comparative genomic hybridization analysis. The black, red, and green dots denote signals indicative of the normal, increased (> +0.5) and decreased (< −1.0) copy‐numbers, respectively. The blue and red boxes represent previously reported XY sex reversal region (XYSR) (Kim et al. 2015) and *SOX9* exons, respectively. Genomic positions refer to the UCSC database (http://genome.ucsc.edu/; GRCh37/hg19). Lower panel: sequence of the fusion junction. The junction is accompanied by a short‐nucleotide insertion of unknown origin (the red‐shaded area).

We identified two heterozygous missense substitutions c.1180C>G (p.Arg394Gly) and c.1309C>T (p.Arg437Cys) in patients 1 and 2, respectively, in *SOX9* (Fig. 1). These substitutions have not been reported previously. Patients 1 and 2 carried no mutations in the other genes examined or in other nucleotides of *SOX9*. The substitution of patient 2 was shared by the phenotypically normal mother, whereas parental samples of patient 1 were not available for genetic analysis. The p.Arg394Gly and p.Arg437Cys substitutions resided within the proline/glutamine/serine (PQS)‐rich domain (also known as SPQ‐rich domain) at the C‐terminus (McDowall et al. 1999) (Fig. 1A).

### Functional analysis of *SOX9* substitutions

The c.1180C>G (p.Arg394Gly) and c.1309C>T (p.Arg437Cys) substitutions involved highly conserved amino acids, and were predicted as “probably damaging” by in silico analyses (Fig. 1B). These substitutions were absent from ~120,000 alleles of the exome database. The p.Arg394Gly and p.Arg437Cys mutants retained normal in vitro transactivating activity for the *Col2a1* enhancer (relative fold activation: 1.14 and 1.17, respectively), but exerted impaired activity for the *Amh* promoter (relative fold activation: 0.74 and 0.81, respectively) (Fig. 1C). In contrast, the previously reported ACD‐associated p.Pro176Leu mutant showed markedly reduced activity for both reporters (relative fold activation: 0.50 for the *Col2al* enhancer and 0.26 for the *Amh* promoter).

### Copy‐number analysis

**Figure 2:**
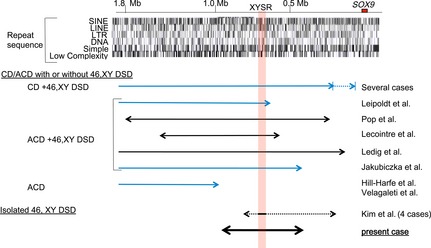
Schematic representation of the *SOX9* upstream region. Upper panel represents positions of *SOX9* and repeat sequences (UCSC database, http://genome.ucsc.edu/; GRCh37/hg19). The numbers indicate the distance from *SOX9*. Lower panel represents genomic rearrangements in the present and previous cases. Blue and black arrows indicate chromosomal translocations and deletions, respectively. Broken arrows indicate breakpoint regions of multiple patients. The red‐shaded area represents the XYSR reported by Kim et al. (2015). XYSR, XY sex reversal region; CD, campomelic dysplasia; ACD, acampomelic CD; DSD, disorders of sex development.

We identified a heterozygous deletion in the upstream region of *SOX9* in patient 3 (Fig. 1D). The deletion was 490,990 bp in physical length and started at a position 380,011 bp upstream to the *SOX9* start codon (chr17: 69,246,534–69,737,523; GRCh37/hg19). The deletion encompassed the known XYSR (Figs. 1D, 2). The deletion breakpoints resided in nonrepeat sequences and shared no homology (Fig. 1D). The fusion junction was accompanied by a short‐nucleotide insertion of unknown origin that was indicative of an “information scar” of nonhomologous end‐joining (NHEJ) (Lieber 2008). Patient 3 carried no sequence alteration in all genes examined. The *SOX9* upstream deletion was identified in the phenotypically normal mother of patient 3.

### Clinical features of the mutation‐positive patients

Physical and hormonal findings of patients 1–3 are summarized in Table 1. Blood inhibin B levels were not determined in these cases. Patient 1 was a 19‐year‐old individual raised as a male. He manifested hypospadias and bilateral cryptorchidism at birth and underwent surgical intervention at 5 years of age. At 14 years of age, he was subjected to endocrine evaluation because of a lack of pubertal sexual development. Blood examinations revealed increased levels of gonadotropins and mildly decreased levels of testosterone, indicating testicular dysfunction. He began to receive testosterone supplementation therapy at age 14, and human chorionic gonadotropin and human menopausal gonadotropin therapy at age 16. He underwent surgical intervention for gynecomastia at 14 and 16 years of age. Abdominal ultrasound at 18 years of age showed small testes with focal microlithiasis. Müllerian duct derivatives were absent. He had no skeletal abnormalities except for spina bifida occulta, a relatively common neural tube anomaly of that has not been associated with *SOX9* mutations (Greene and Copp 2014).

**Table 1:** Molecular and clinical findings of patients 1–3

	Patient 1		Patient 2		Patient 3	
Karyotype	46,XY		46,XY		46,XY	
Molecular defects in *SOX9* (NM_000346.3)	c.1180C>G (p.Arg394Gly)		c.1309C>T (p.Arg437Cys)		Upstream deletion	
Physical findings at birth						
External genitalia	Male‐type genitalia with hypospadias and unpalpable testes		Male‐type genitalia with micropenis and unpalpable testes		Complete female‐type genitalia	
Physical findings at later ages						
Age at exam. (year)	19		2.8		22	
Penile size (cm)^a^	**5.7** b (8.6–10.0)		**2.5** (3.0–3.6)		Not examined	
Testicular size (mL)	5 (right), 3–4 (left)^c^		Not palpable		Not palpable	
Gonadal histology	Not analyzed		Fibrous tissues		Streak gonad with seminoma	
Uterus	Absent		Absent		Present	
Additional findings	Spina bifida occulta		None		None	
Hormonal findings^a^						
Age at exam. (year)	19		2.8		13	
	B	S	B	S	B	S
LH (mIU/mL)^d^	*37.5* (0.5–5.0)	*126.6* (6.0–21.0)	*2.8* (<0.3–1.3)	Not analyzed	*13.8* (<0.2–2.1)	*119.4* (1.3–7.0)
FSH (mIU/mL)^d^	*17.7* (0.8–4.4)	*24.3* (1.5–8.0)	*109.8* (0.4–1.5)	Not analyzed	*82.5* (<0.3–3.0)	*135.8* (1.3–3.9)
Testosterone (ng/mL)^e^	5.3 (2.5–11.0)	**4.9** (6.3–9.8)	**<0.03** (0.06–0.16)	**<0.03** (>0.2)	**0.09** (0.68–1.22)	**0.16** (2.96–5.58)
AMH (ng/mL)	Not analyzed	Not analyzed	**<0.1** (74.1–148.1)	<0.1 (no data)	Not analyzed	Not analyzed

The conversion factor to the SI unit: LH 1.0 (IU/L), FSH 1.0 (IU/L), testosterone 3.47 (nmol/L), and AMH 7.14 (pmol/L). Penile size and hormone values below the reference range are boldfaced, and hormone values above the reference range are italicized. B, basal; S, stimulated; LH, luteinizing hormone; FSH, follicle‐stimulating hormone; AMH, anti‐Müllerian hormone.

a Reference ranges are shown in parentheses.

b After hormone replacement therapy.

c After surgical interventions.

d Gonadotropin releasing hormone stimulation test (100 *μ*g/m^2^, max. 100 *μ*g bolus i.v.; blood sampling at 0, 30, 60, 90, and 120 min).

e Human chorionic gonadotropin stimulation test (3000 IU/m^2^, max. 5000 IU i.m. for three consecutive days; blood sampling on days 1 and 4).

Patient 2 was a 5‐year‐old male individual. At birth, he showed male‐type external genitalia; however, bilateral testes were not palpable in the scrotum or in the inguinal region. At 2.6 years of age, laparoscopic examination detected possible gonadal remnants with spermatic cord in the bilateral inguinal canals. Histological examination of biopsied samples showed that the possible remnants were fibrous tissues without germ cells. At 2.8 years of age, he was referred to our clinic for further evaluation. He showed normal skeletal features and borderline micropenis. Endocrine analyses revealed increased gonadotropin levels and undetectable levels of testosterone and anti‐Müllerian hormone. Abdominal imaging did not detect a uterus or gonads. Thus, this patient was diagnosed with testicular regression. At 5 years of age, he was capable of voiding in a standing position.

Patient 3 was a 22‐year‐old individual with a female phenotype. Her growth and development were uneventful until pubertal age. At 13 years of age, she visited our clinic because of a lack of pubertal sexual development. She showed normal skeletal features and female‐type external genitalia. Endocrine evaluation indicated severe gonadal dysfunction. Abdominal magnetic resonance imaging detected a uterus of a prepubertal size. Estrogen supplementation therapy from 14 years of age successfully induced breast budding and vaginal bleeding. Gonadectomy was performed at 17 years of age. Histological examination revealed bilateral dysgenic gonads with seminoma.

## Discussion

This study provides several notable findings. This is the first report documenting the association between *SOX9* intragenic mutations and isolated 46,XY DSD. In vitro assays confirmed the target‐specific functional impairment of the c.1180C>G (p.Arg394Gly) and c.1309C>T (p.Arg437Cys) mutants, which were not observed in the known ACD‐associated mutant c.527C>T (p.Pro176Leu). Notably, unlike other known pathogenic *SOX9* missense mutations, p.Arg394Gly and p.Arg437Cys resided within the C‐terminal PQS‐rich domain. As the PQS‐rich domain is required for SOX9 interaction with other proteins (Tsuda et al. 2003), the two mutations may affect SOX9‐mediated protein–protein interactions in the developing testis. Physical and hormonal findings of patients 1 and 2 with these mutations were indicative of impaired testicular development, although blood inhibin B levels, a sensitive marker for the function of the testis (Grinspon et al. 2012), were not determined in these patients.

It is worth mentioning that patient 2 manifested bilateral testicular regression, a rare form of 46,XY DSD that probably occurs as a result of the disturbance of developmental processes during testicular tubule formation (Mizuno et al. 2012). The genetic basis of testicular regression remains unknown, with the exception of *NR5A1* mutations that account for a minor fraction of cases (Philibert et al. 2007). It has been suggested that testicular regression and gonadal dysgenesis are a continuum of a disorder (Marcantonio et al. 1994). Indeed, *NR5A1* mutations are known to underlie both conditions (Ferraz‐de‐Souza et al. 2011). Animal studies suggested that SOX9 plays a role in testicular tubule differentiation through the interaction with SOX8 (Barrionuevo et al. 2009). Moreover, SOX9 plays a critical role not only in testicular development but also in the maintenance of differentiated status of the testes (Sekido and Lovell‐Badge 2009; Veitia 2010). Thus, testicular regression may be a rare manifestation in patients with *SOX9* mutations. However, this notion is based on the findings of a single individual, and therefore awaits further investigation.

One may argue against p.Arg394Gly and p.Arg437Cys being responsible for the severe DSD in patients 1 and 2 because these mutations resulted in only modest decrease in the transactivating activity for the *AMH* promoter. The discrepancy between the phenotypic severities and the results of in vitro assays can be explained by assuming that some SOX9 target genes other than *AMH* are more sensitive to defective function of SOX9. Actually, a number of testicular genes are known to be regulated by SOX9 (Sekido and Lovell‐Badge 2009; Veitia 2010). Alternatively, in the developing testis, the p.Arg394Gly and p.Arg437Cys mutations may disrupt the synergic interaction between SOX9 and certain cofactors. It is known that SOX9 synergizes with other proteins to transactivate target genes (Sekido and Lovell‐Badge 2009; Veitia 2010). On the other hand, we cannot exclude the possibility that genetic variations in other genes or some environmental factors affected sexual development in patients 1 and 2, although mutations in known 46,XY DSD causative genes were excluded in these patients. Further studies, including in vitro assays using reporter vectors containing various SOX9 target promoters and expression vectors for several SOX9 cofactors, and whole exome sequencing of patients 1 and 2, will clarify the precise functional consequences of the mutants.

The findings in patient 3 support the notion that the 32.5 kb XYSR contains a DNA element(s) essential for testicular development. Moreover, exclusion mapping indicates that *SOX9* enhancers for skeletal tissues and craniofacial regions are located outside of the ~491 kb region deleted in patient 3 (Fig. 2) (Pop et al. 2004; Hill‐Harfe et al. 2005; Velagaleti et al. 2005; Lecointre et al. 2009; Jakubiczka et al. 2010; Ledig et al. 2010; Kim et al. 2015). In addition, the results of this study, together with those of previous studies (Pop et al. 2004; Lecointre et al. 2009; Kim et al. 2015) provide evidence of the genomic heterogeneity of *SOX9* upstream deletions. The breakpoint sequences in patient 3 suggest that the deletion resulted from NHEJ. Actually, genomic regions around *SOX9* are not enriched with repeat sequences that serve as substances of nonallelic homologous recombination (Fig. 2). Thus, NHEJ may be the major cause of *SOX9* upstream microdeletions, although other mechanisms such as microhomology‐mediated replication errors may also be involved in the development of such deletions. Since all known pathogenic deletions in the *SOX9* upstream region, including that in our patient, were transmitted from phenotypically normal mothers of the patients (Kim et al. 2015), de novo occurrence of such deletions seems to be a rare event.

In summary, the results indicate that the phenotypic consequences of *SOX9* mutations are broader than previously reported and include testicular dysgenesis and regression without skeletal dysplasia. Furthermore, our data suggest that DSD‐associated *SOX9* abnormalities include C‐terminal missense substitutions that lead to target‐specific protein dysfunction, and NHEJ‐mediated upstream microdeletions encompassing XYSR.

## Conflict of Interest

None declared.
